# Prognosis comparison between intraoperative radiotherapy and whole-breast external beam radiotherapy for T1–2 stage breast cancer without lymph node metastasis treated with breast-conserving surgery: A case–control study after propensity score matching

**DOI:** 10.3389/fmed.2022.919406

**Published:** 2022-08-03

**Authors:** Qitong Chen, Limeng Qu, Yeqing He, Jiachi Xu, Yueqiong Deng, Qin Zhou, Wenjun Yi

**Affiliations:** Department of General Surgery, The Second Xiangya Hospital of Central South University, Changsha, China

**Keywords:** breast cancer, intraoperative radiotherapy (IORT), propensity score matching (PSM), the Surveillance, Epidemiology, and End Results (SEER), T1-2 stage

## Abstract

**Background:**

External beam radiotherapy (EBRT), an adjuvant to breast-conserving surgery (BCS), requires a long treatment period, is costly, and is associated with numerous complications. Large sample studies with long follow-up periods are lacking regarding whether intraoperative radiotherapy (IORT), an emerging radiotherapy modality, can replace EBRT for patients with T1–2 early stage breast cancer without lymph node metastasis treated with BCS.

**Methods:**

We identified 270,842 patients with T1-2N0M0 breast cancer from 2000 to 2018 in the Surveillance, Epidemiology, and End Results (SEER) database. A total of 10,992 patients were matched by propensity score matching (PSM). According to the radiotherapy method, the patients were divided into the IORT and EBRT groups. Overall survival (OS) and breast cancer-specific survival (BCSS) rates were analyzed and compared between the IORT and EBRT groups by Kaplan–Meier analysis. Bilateral *P* < 0.05 was considered to indicate significance.

**Results:**

After PSM, the survival analysis showed no significant differences in OS or BCSS rates between the IORT and EBRT groups. In the subgroup analysis, the IORT population diagnosed from 2010 to 2013 (HRs = 0.675, 95% CI 0.467–0.976, *P* = 0.037) or with T2 stage (HRs = 0.449, 95% CI 0.261–0.772, *P* = 0.004) had better OS rates, but in the overall population, the OS and BCSS rates were better in patients with T1 stage than in patients with T2 stage (*P* < 0.0001), and the proportion of chemotherapy was significantly higher in T2 stage than in T1 stage. Patients who had EBRT with unknown estrogen receptor had better OS rates (HRs = 3.392, 95% CI 1.368–8.407, *P* = 0.008). In addition, the IORT group had better BCSS rates for married (HRs = 0.403, 95% CI 0.184–0.881, *P* = 0.023), grade III (HRs = 0.405, 95% CI 0.173–0.952, *P* = 0.038), and chemotherapy-receiving (HRs = 0.327, 95% CI 0.116–0.917, *P* = 0.034) patients with breast cancer compared to the EBRT group.

**Conclusion:**

Intraoperative radiotherapy results of non-inferior OS and BCSS rates, compared to those of EBRT, in patients with early stage breast cancer without lymph node metastasis treated with BCS, and IORT may provide substantial benefits to patients as an effective alternative to standard treatment. This finding provides new insights into radiotherapy strategies for early stage breast cancer.

## Introduction

Breast cancer is the most common cancer in women and the main cause of cancer-related death (1). Breast-conserving surgery (BCS) has become the preferred option for patients with early stage breast cancer, and standard adjuvant radiation therapy can greatly reduce the risk of recurrence and improve survival rates (2–4). The standard mode of post-BCS radiotherapy is whole-breast external beam radiotherapy (EBRT), and conventionally fractionated whole breast irradiation was generally conducted consisting of 50 Gy in 25 fractions with or without a tumor bed boost and delivered over the course of 5–7 weeks, while hypofractionated whole breast irradiation reduces the treatment time to 3–4 weeks. On the whole, EBRT reduced patient compliance with treatment, caused skin hyperpigmentation and atrophy, and damage to organs near the irradiated site (5–7). In an analysis of recurrence patterns in patients with breast cancer after BCS, it was found that 90% of recurrences after BCS were concentrated in the quadrant where the lesion was located and that the recurrence rate of breast cancer in areas other than the ipsilateral breast tumor bed was similar to the incidence of contralateral second primary breast cancer (8–10). Therefore, accelerated partial breast irradiation (APBI) is gradually being used to replace EBRT, increasing the dose given in a single treatment and reducing the area and duration of exposure, and it has become the treatment option listed by the National Comprehensive Cancer Network guidelines (11, 12). Intraoperative radiotherapy (IORT) is a form of APBI radiotherapy first used in the 1960s. IORT is a single high-dose radiotherapy treatment for the tumor bed, residual lesions, and lymphatic drainage areas directly observed during surgery; it has the advantages of shortening treatment duration and effectively protecting normal tissues and has been used for low-risk patient groups, especially those with early stage breast cancer without axillary lymph node metastasis (13–17).

The impact of IORT on breast cancer prognosis is being continuously explored. Many studies to date have shown that IORT is non-inferior to EBRT in terms of OS rates for BCS of early stage breast cancer (18–24). The TARGIT-A study compared immediate targeted intraoperative radiotherapy with EBRT, and a long-term follow-up study revealed that there were no statistically significant differences in local recurrence-free survival, mastectomy-free survival, distant disease-free survival, overall survival, or breast cancer mortality rates, and that the risk for non-tumor-related death was significantly lower; however, non-inferiority for local recurrence could not be demonstrated in the post-pathology cohort, and approximately 15% of patients in the IORT group received supplemental EBRT (25, 26). However, the ELIOT study compared electron beam intraoperative radiotherapy (ELIOT) and whole breast irradiation (WBI) and found that the 5-year OS rates were similar, but that the rates of regional lymph node metastasis and local recurrence were higher in the ELIOT group than in the EBRT group (19, 27). These studies all had strict enrollment criteria; more than 70% of the patients had T1 stage and HR-positive breast cancer, and all of the studies were conducted in the 2000s. The long-term survival benefit of IORT needs to be further explored because of changes in adjuvant treatment modalities, such as the prevalence of SLNB surgery, more active radiotherapy indications, and more precise adjuvant treatment modes.

Therefore, our study aimed to assess the long-term prognostic and survival benefits of IORT in women with breast cancer without lymph node metastases at stage T1-2 based on data from the Surveillance, Epidemiology, and End Results (SEER) database. We conducted a retrospective study on data from 270,842 people diagnosed with early stage breast cancer (T1-2N0M0) between 2000 and 2018, applying statistical methods, such as propensity score matching (PSM) and Cox analysis models to control for selection bias, and confounding by balancing confounders. This study provides a large sample-based exploration of the long-term survival benefit of IORT vs. EBRT in patients with early stage breast cancer without lymph node metastases and further demonstrates the safety and efficacy of IORT.

## Materials and methods

### Data source and cohort selection

The SEER database registry program supported by the National Cancer Institute currently collects and provides information on cancer incidence from population-based cancer registries covering approximately 48% of the United States population (28). The demographic, clinicopathological, tumor morphology, treatment and vital information data were acquired from the SEER program^1^ *via* the SEER*Stat software (version 8.3.9.2)^2^ in a client-server model with permission from the SEER program office. The study followed the Strengthening the Reporting of Observational Studies in Epidemiology (STROBE) reporting guidelines (29). Since the SEER database is public and de-identified, this study was deemed exempt from review by the Ethics Committee of the Second Xiangya Hospital of Central South University.

**FIGURE 1:**
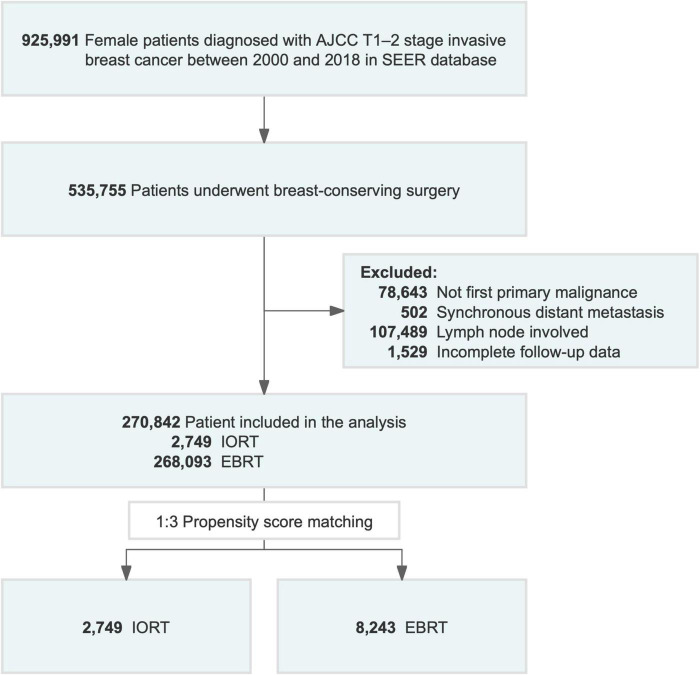
Flowchart of patient selection for the study. AJCC, American Joint Committee on Cancer; EBRT, external beam radiotherapy; IORT, intraoperative radiotherapy; SEER, Surveillance, Epidemiology, and End Results.

Patients diagnosed with pathologically confirmed breast cancer from 2000 to 2018 were enrolled in the study. Patients were included if they met the following criteria: (1) female, (2) age at diagnosis over 18 years, (3) AJCC T1–2 stage disease, and (4) available information on TNM staging system and (5) not carcinomas *in situ*. The exclusion criteria were as follows:(1) no first primary malignancy, (2) incomplete follow-up data, (3) synchronous distant metastatic disease (M1), and (4) diseases other than AJCC N0 stage disease. All coding rules for data collection were specified in the SEER program coding and staging manual (30). Ultimately, 270,842 female patients (IORT [*n* = 2,749] vs. EBRT [*n* = 268,093]) with primary breast cancer without lymph node involvement or distant metastases were selected. The flow chart of the patient selection process is presented in Figure 1.

### Variables

The following demographic, clinicopathologic characteristic, and treatment information on patients with T1–T2N0M0 breast cancer (Table 1) was included: age at diagnosis, marital status, race, year of diagnosis, grade, breast-adjusted T stage based on the AJCC-TNM stage system, estrogen receptor (ER) status, progesterone receptor (PR) status, HER2 (human epidermal growth factor receptor 2) status, molecular subtype, and chemotherapy status. The SEER database started to document HER2 status data from January 2010; thus, HER2 status information was unavailable for some of the patients enrolled in this study (31). Continuous variables and age at diagnosis were transformed into categorical variables (≤45, 46–65, and >65). Analyses of survival (months), vital status, and cause-specific death classification were performed to evaluate prognostic outcomes.

**TABLE 1:** Characteristics of female patients diagnosed with primary T1-T2N0M0 breast cancer in Surveillance, Epidemiology, and End Results (SEER) database.

Variables	Before propensity score matching, n (%)				After propensity score matching, n (%)			
	Overall	IORT	EBRT	*P*-value	Overall	IORT	EBRT	*P*-value
Age				<0.001				0.925
≤45	26372 (9.7)	70 (2.5)	26302 (9.8)		288 (2.6)	70 (2.5)	218 (2.6)	
46–65	145687 (53.8)	1347 (49.0)	144340 (53.8)		5407 (49.2)	1347 (49.0)	4060 (49.3)	
>65	98783 (36.5)	1332 (48.5)	97451 (36.3)		5297 (48.2)	1332 (48.5)	3965 (48.1)	
Year				<0.001				0.998
2000–2004	60676 (22.4)	129 (4.7)	60547 (22.6)		523 (4.8)	129 (4.7)	394 (4.8)	
2005–2009	66274 (24.5)	96 (3.5)	66178 (24.7)		383 (3.5)	96 (3.5)	287 (3.5)	
2010–2013	57785 (21.3)	622 (22.6)	57163 (21.3)		2479 (22.6)	622 (22.6)	1857 (22.5)	
2014–2018	86107 (31.8)	1902 (69.2)	84205 (31.4)		7607 (69.2)	1902 (69.2)	5705 (69.2)	
Marital status				<0.001				0.920
Married	163126 (60.2)	1608 (58.5)	161518 (60.2)		6458 (58.8)	1608 (58.5)	4850 (58.8)	
Single	33083 (12.2)	441 (16.0)	32642 (12.2)		1752 (15.9)	441 (16.0)	1311 (15.9)	
DSW	64857 (23.9)	610 (22.2)	64247 (24.0)		2443 (22.2)	610 (22.2)	1833 (22.2)	
Unknown	9776 (3.6)	90 (3.3)	9686 (3.6)		339 (3.1)	90 (3.3)	249 (3.0)	
Race				<0.001				0.552
White	223987 (82.7)	2250 (81.8)	221737 (82.7)		9030 (82.2)	2250 (81.8)	6780 (82.3)	
African American	23443 (8.7)	196 (7.1)	23247 (8.7)		789 (7.2)	196 (7.1)	593 (7.2)	
Other	22288 (8.2)	277 (10.1)	22011 (8.2)		1091 (9.9)	277 (10.1)	814 (9.9)	
Unknown	1124 (0.4)	26 (0.9)	1098 (0.4)		82 (0.7)	26 (0.9)	56 (0.7)	
Histology				<0.001				0.963
Ductal carcinoma	205702 (75.9)	2189 (79.6)	203513 (75.9)		8734 (79.5)	2189 (79.6)	6545 (79.4)	
Lobular carcinoma	35380 (13.1)	285 (10.4)	35095 (13.1)		1145 (10.4)	285 (10.4)	860 (10.4)	
Other	29760 (11.0)	275 (10.0)	29485 (11.0)		1113 (10.1)	275 (10.0)	838 (10.2)	
Grade				<0.001				0.791
I	79999 (29.5)	1109 (40.3)	78890 (29.4)		4453 (40.5)	1109 (40.3)	3344 (40.6)	
II	114190 (42.2)	1255 (45.7)	112935 (42.1)		5032 (45.8)	1255 (45.7)	3777 (45.8)	
III	63358 (23.4)	344 (12.5)	63014 (23.5)		1356 (12.3)	344 (12.5)	1012 (12.3)	
IV	1395 (0.5)	3 (0.1)	1392 (0.5)		18 (0.2)	3 (0.1)	15 (0.2)	
Unknown	11900 (4.4)	38 (1.4)	11862 (4.4)		133 (1.2)	38 (1.4)	95 (1.2)	
T stage				<0.001				1.000
T1	221382 (81.7)	2407 (87.6)	218975 (81.7)		9626 (87.6)	2407 (87.6)	7219 (87.6)	
T2	49460 (18.3)	342 (12.4)	49118 (18.3)		1366 (12.4)	342 (12.4)	1024 (12.4)	
ER				<0.001				0.984
Positive	224278 (82.8)	2587 (94.1)	221691 (82.7)		10350 (94.2)	2587 (94.1)	7763 (94.2)	
Negative	38092 (14.1)	138 (5.0)	37954 (14.2)		545 (5.0)	138 (5.0)	407 (4.9)	
Unknown	8472 (3.1)	24 (0.9)	8448 (3.2)		97 (0.9)	24 (0.9)	73 (0.9)	
PR				<0.001				0.843
Positive	196087 (72.4)	2373 (86.3)	193714 (72.3)		9497 (86.4)	2373 (86.3)	7124 (86.4)	
Negative	63872 (23.6)	349 (12.7)	63523 (23.7)		1397 (12.7)	349 (12.7)	1048 (12.7)	
Unknown	10883 (4.0)	27 (1.0)	10856 (4.0)		98 (0.9)	27 (1.0)	71 (0.9)	
HER2				<0.001				0.794
Positive	14470 (5.3)	111 (4.0)	14359 (5.4)		416 (3.8)	111 (4.0)	305 (3.7)	
Negative	123784 (45.7)	2347 (85.4)	121437 (45.3)		9420 (85.7)	2347 (85.4)	7073 (85.8)	
Unknown	5571 (2.1)	66 (2.4)	5505 (2.1)		248 (2.3)	66 (2.4)	182 (2.2)	
Unavailable	127017 (46.9)	225 (8.2)	126792 (47.3)		908 (8.3)	225 (8.2)	683 (8.3)	
Molecular Subtype				<0.001				0.945
HR + /HER2-	111265 (41.1)	2275 (82.8)	108990 (40.7)		9138 (83.1)	2275 (82.8)	6863 (83.3)	
HR + /HER2 +	10933 (4.0)	96 (3.5)	10837 (4.0)		358 (3.3)	96 (3.5)	262 (3.2)	
HER2 enriched	3515 (1.3)	15 (0.5)	3500 (1.3)		58 (0.5)	15 (0.5)	43 (0.5)	
TNBC	12410 (4.6)	71 (2.6)	12339 (4.6)		280 (2.5)	71 (2.6)	209 (2.5)	
Unknown	132719 (49.0)	292 (10.6)	132427 (49.4)		1158 (10.5)	292 (10.6)	866 (10.5)	
Chemotherapy				<0.001				0.794
Chemotherapy	72158 (26.6)	280 (10.2)	71878 (26.8)		1136 (10.3)	280 (10.2)	856 (10.4)	
Chemotherapy-naïve	198684 (73.4)	2469 (89.8)	196215 (73.2)		9856 (89.7)	2469 (89.8)	7387 (89.6)	
**Total**	270842	2749	268093		10992	2749	8243	

DSW, divorced/separated/widowed; EBRT, external beam radiotherapy; ER, estrogen receptor; HER2, human epidermal growth receptor 2; HR, hormone receptor; IORT, intraoperative radiotherapy; PR, progesterone receptor; TNBC, triple-negative breast cancer.

### Propensity score matching

Paul R. Rosenbaum and Donald Rubin introduced the propensity score technique in 1983 (32). The propensity score is a balancing score: conditional on the propensity score, the distribution of measured baseline covariates is similar between treated and untreated subjects. Propensity score matching allow for one to mimic some of the characteristics of a randomized controlled trial (RCT) in the context of an observational study. This study was a retrospective case–control study (non-randomized), and some variables were potential confounders of treatment effect, such as age, marital status, race, year of diagnosis, grade, T stage, histology, estrogen receptor (ER) status, progesterone receptor (PR) status, human epidermal growth receptor 2 (HER2) status, molecular subtype, and chemotherapy, exhibited heterogeneity between the IORT and EBRT patients in the SEER database (Table 1). PSM is a reliable statistical method that can control selection bias and balance covariates affecting prognosis in non-randomized studies (33). We implemented PSM (34) using R package “MatchIt” (35) version 4.1.0 with the following settings: 1:3 pairing, nearest-neighbor methods, and a caliper of 0.1 to balance the baseline characteristics of patients treated with IORT or EBRT. After PSM, the demographic and clinicopathological characteristics of the patients with breast cancer were well balanced and included in further analyses. The patients were divided into the following main subgroups: IORT and EBRT.

### Statistical analyses

Pearson’s χ2 test was conducted to assess the heterogeneity of categorical variables between the IORT and EBRT groups. Overall survival (OS) was defined as the time from diagnosis to death from any cause, and BCSS was defined as the time from the initial diagnosis to breast cancer-related death. OS and BCSS rates were the primary endpoints of this study. Survival curves for OS and BCSS rates were constructed using the Kaplan–Meier methodology (36). Hazard ratios (HRs) and 95% confidence intervals (CIs) for OS and BCSS rates between the IORT and EBRT interventions were estimated by a univariable Cox proportional hazards regression analysis [using the R package “survminer” (37)] and are presented in forest plots (using the R package “forestplot”). Statistical analyses and data visualization were performed using R (version 4.1.2)^3^ and RStudio (R-Studio Inc., Boston, United States, version 1.4.1103). All the statistical tests were two-sided, and statistical significance level was set at *P* < 0.05.

## Results

### Baseline characteristics

**FIGURE 2:**
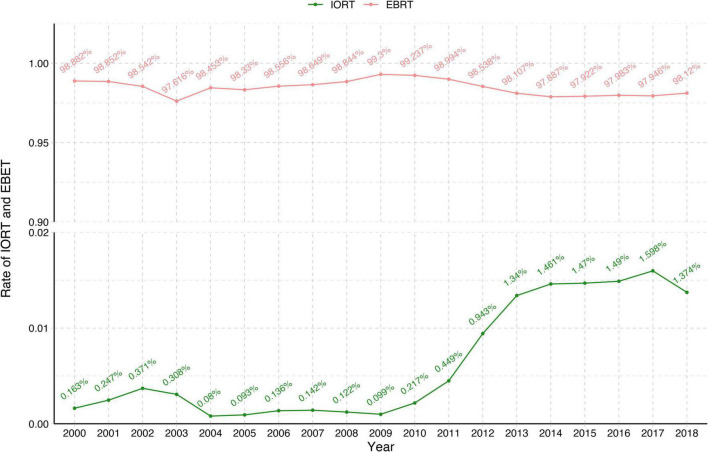
Changes in the rates of IORT and EBRT from 2000 to 2018. EBRT, external beam radiotherapy; IORT, intraoperative radiotherapy.

Between 2000 and 2018, 270,842 female patients with T1–2N0M0 breast cancer in the SEER database who underwent BCS received IORT (2,749, 1.01%) or EBRT (268,093, 98.9%) (Figure 1 and Table 1). We visually assessed changes in the incidence of IORT and EBRT applications between 2000 and 2018. The use of IORT increased gradually from 0.163% in 2000 to 1.374% in 2018, while the use of EBRT began to decline slightly in 2010 (Figure 2).

The median age of the eligible patients was 62 years (range 18–99 years). The median follow-up time was 81 months. Most of the patients treated with IORT were 46–65 years old (1,347, 49%), diagnosed in 2014–2018 (1,902, 69.2%), and married (1,608, 58.5%). Most of the patients treated with IORT were white (2,250, 81.8%), a small number (196, 7.1%) was African American, and the rest were of a different race (277, 10.1%). Grades I–II (1,109, 62.6% and 1,255, 45.7%). were reported in most patients who received IORT. A total of 2,407 (87.6%) patients had stage T1 disease, and 342 (12.4%) patients had stage T2 disease. The histological type was ductal carcinoma in 79.6% (2,189/2,749) of the patients. A total of 94.1% of the patients were ER-positive, 86.3% were PR-positive, and 4% were HER2-enriched. HR + /HER2- (2,275, 92.6%) was the most common molecular subtype among the patients with available data. Only 10.2% of the patients received adjuvant chemotherapy. Most of them did not receive chemotherapy (Table 1).

There was heterogeneity in the variables between the IORT and EBRT groups in the initial cohort. Following PSM, a total of 10,992 patients (IORT *n* = 2,749 vs. EBRT *n* = 8,243) were selected for the propensity score-matched cohort. All variables were adequately balanced between the two groups (Table 1). The baseline characteristics of the patients before and after propensity score matching are summarized in Table 1.

### Analysis of survival benefit comparison between intraoperative radiotherapy and external beam radiotherapy

**FIGURE 3:**
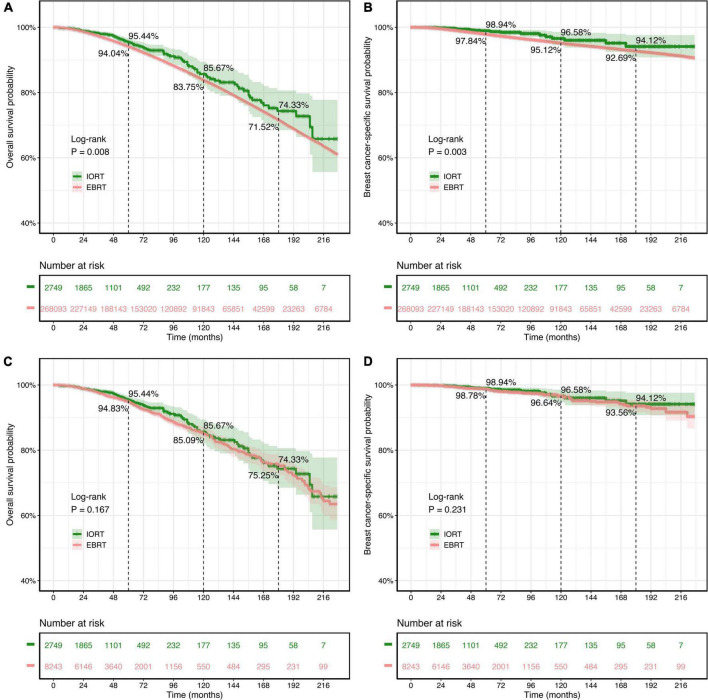
Kaplan–Meier survival curves of overall survival (OS) and breast cancer-specific survival (BCSS) rates for patients with T1–2N0M0 breast cancer treated with IORT or EBRT. **(A,B)** OS and BCSS rates of the original cohort. **(C,D)** OS and BCSS rates of the PSM cohort. Log-rank tests determined the *P*-values. EBRT, external beam radiotherapy; IORT, intraoperative radiotherapy; PSM, propensity score matching.

The survival analyses showed that patients with breast cancer who underwent BCS but did not receive radiation had worse OS and BCSS rates (*P* < 0.0001, Supplementary Figure 1). In the initial cohort, it was noted that the 5-year (95.44% vs. 94.04%; 98.94% vs. 97.84%), 10-year (85.67% vs. 83.75%; 96.58% vs. 95.12%), and 15-year (74.33% vs. 71.52%; 94.12% vs. 92.69%) OS and BCSS rates of the IORT group were higher than those of the EBRT group (Figures 3A,B). Kaplan–Meier survival curves and the log-rank test indicated that OS (*P* = 0.008, Figure 3A) and BCSS (*P* = 0.003, Figure 3B) intervals were longer in the IORT group than in the EBRT group in the cohort before PSM.

The findings above were not present in the survival analysis of the PSM cohort. There was no significant difference in OS (*P* = 0.167) or BCSS (*P* = 0.231) rates between the IORT and EBRT groups according to the log-rank test (Figures 3C,D). In the PSM cohort, in which all variables were well balanced, both the IORT and EBRT populations had similar 5-year (95.44% vs. 94.83%), 10-year (85.67% vs. 85.09%), and 15-year (74.33% vs. 75.25%) OS rates, and similar results were observed regarding BCSS, with comparable 5-year (98.94% vs. 98.78%), 10-year (96.58% vs. 96.64%), and 15-year (94.12% vs. 93.56%) BCSS rates in the IORT and EBRT populations.

### Subgroup analysis

**FIGURE 4:**
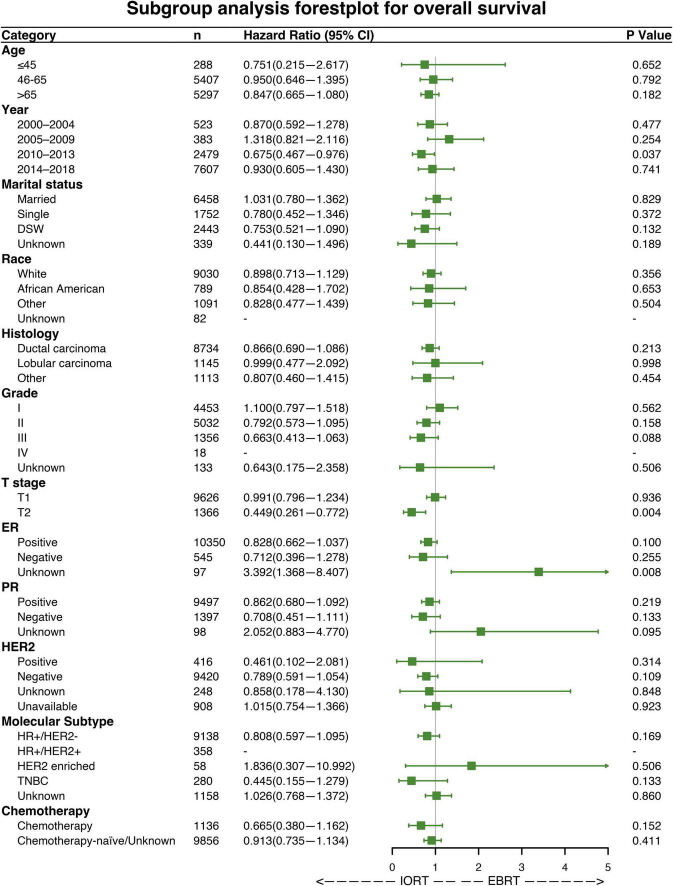
Forest plot of patients with breast cancer in the subgroup analysis (IORT vs. EBRT). Hazard ratios (HRs) with 95% confidence intervals (CIs) for death in terms of the overall survival (OS) rate of patients with breast cancer who underwent IORT or EBRT. *P*-values of the Cox proportional hazards regression are reported. EBRT, external beam radiotherapy; IORT, intraoperative radiotherapy.

**FIGURE 5:**
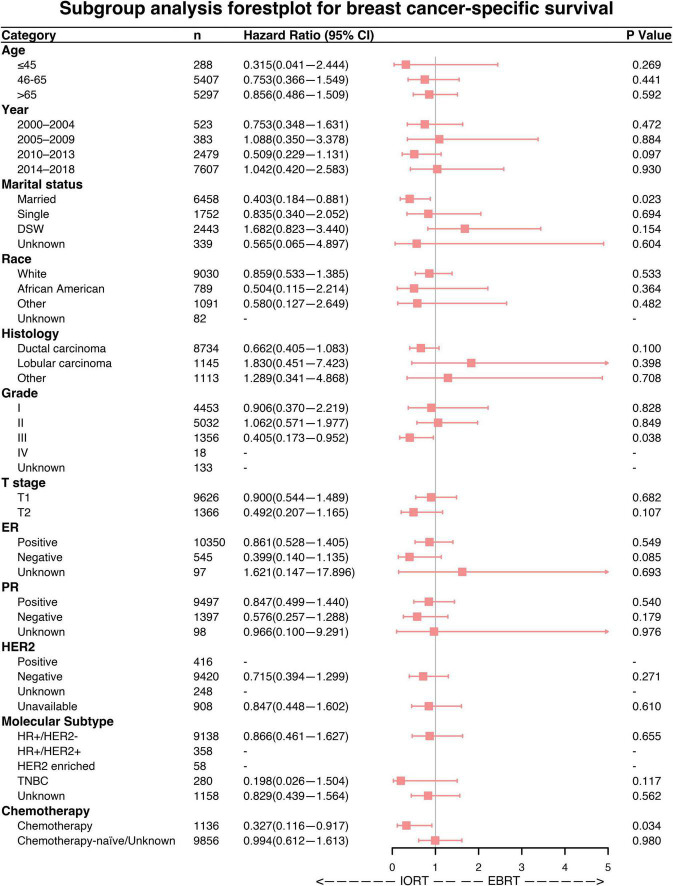
Forest plot of breast cancer patients in the subgroup analysis (IORT vs. EBRT). Hazard ratios (HRs) with 95% confidence intervals (CIs) for death in terms of breast cancer-specific survival (BCSS) of patients with breast cancer who underwent IORT or EBRT. *P*-values of the Cox proportional hazards regression are reported. EBRT, external beam radiotherapy; IORT, intraoperative radiotherapy.

Subgroup analyses were performed to investigate the efficacy of different radiation sequences on OS and BCSS rates. Forest plots of the subgroup analysis are shown in Figures 4, 5. The univariable Cox analysis, including the IORT and EBRT groups, revealed similar outcomes. Most of the subgroups showed no significant OS rate differences (Figure 4) except for year of diagnosis (2010–2013; HRs = 0.675, 95% CI 0.467–0.976, *P* = 0.037) and T2 stage (HRs = 0.449, 95% CI 0.261–0.772, *P* = 0.004). Further survival analysis of T stage in the PSM population revealed that the OS and BCSS rates were higher in the T1-stage population than in the T2-stage population (*P* < 0.0001, Supplementary Figure 2). Moreover, in the T2-stage subset of the population, all variables were balanced between the IORT and EBRT groups (Supplementary Table 2). Similarly, few significant BCSS rate differences were found in the subgroup analyses. However, some of the variables showed that IORT was beneficial for patients with breast cancer compared to EBRT (Figure 5), with marital status (HRs = 0.403, 95% CI 0.184–0.881, *P* = 0.023), grade III differentiation (HRs = 0.405, 95% CI 0.173–0.952, *P* = 0.038), and chemotherapy (HRs = 0.327, 95% CI 0.116–0.917, *P* = 0.034) associated with better BCSS rates. These results may indicate that IORT is non-inferior to EBRT for patients with breast cancer and shows tremendous advantages in some subgroups.

## Discussion

In the past 20 years, many changes have occurred in radiotherapy approaches triggered by the introduction of modern high-precision techniques and simultaneous improvements in our understanding of tumor biology in clinical settings (27). APBI, which involves direct irradiation of breast tissues adjacent to the surgically resected area, stands out as a highly valuable approach. The current APBI technologies are brachytherapy(BT), 3D conformal radiotherapy(3DCRT), intensity-modulated radiotherapy (IMRT), and IORT; among which IORT is the latest (38).

Studies based on the SEER database have a high clinical reference value because of large sample size and strong statistical efficacy. We have previously explored the impact of chemotherapy on the prognosis of patients with metaplastic breast carcinoma (MpBC) and compared the long-term prognosis of nipple-sparing mastectomy (NSM) vs. total mastectomy (TM) by analyzing different populations in the SEER database, providing large sample-based evidence for breast cancer treatment (39, 40).

This study compared survival outcomes between SEER database women with early stage breast cancer without lymph node metastases who received IORT or EBRT. The results of this study showed no significant differences in OS or BCSS rates between the IORT and EBRT populations at 5, 10, or 15 years, which is consistent with the results of the ELIOT study and the TARGIT-A study (25, 27). In addition, in a subgroup analysis of patients with early stage breast cancer, we found that the IORT population with diagnosis years of 2010–2013 had a better OS probably because the rate of IORT showed a substantial increase from 2010 to 2013, while the rate of EBRT started to decrease slightly in 2010 (Figure 2). IORT in patients who were married was associated with better BCSS, which may be related to the baseline characteristics of the enrolled patients, with 97.4% of the study population being older than 45 years. Additionally, in the overall study population, the proportion of married patients (58.8%) was significantly higher than that of patients who were single (15.9%), divorced/separated/widowed (22.2%), or with an unknown status (3.1%). Further analysis of ethnic distribution showed that African Americans represented only 7.2% of the total population, and that there was no prognostic difference between the IORT and EBRT populations for these groups, but further confirmation is needed in large sample studies. We also found that IORT may be associated with better OS rates among patients with tumor stage T2, but in the overall population, patients with tumor stage T1 had better OS and BCSS rates than patients with tumor stage T2, which may be due to a higher incidence of chemotherapy among patients with tumor stage T2 (Supplementary Table 1). Additionally, the IORT population that had received chemotherapy also had a better BCSS rate. The inclusion of patients who received chemotherapy in the population suitable for IORT is still controversial, and ASTROAPBI guidelines do not recommend IORT for patients administered with neoadjuvant chemotherapy. However, one of the advantages of neoadjuvant chemotherapy is that it allows for a subset of patients who do not meet the conditions for breast conservation to undergo BCS while at the same time allowing for the sensitivity of the tumor to chemotherapy to be examined. Pathologic complete response (PCR) is often considered to predict a better prognosis (41, 42), and patients treated with neoadjuvant chemotherapy are at higher risk for local and distant recurrences due to tumor biology (43). This subset of high-risk patients may benefit from the better local disease control achieved with IORT, and in previous studies, the use of IORT in patients who received breast-conserving therapy after neoadjuvant chemotherapy did not affect the cosmetic outcome or interfere with the pathological assessment of incision margins (44–46). Thus, IORT is not inferior to EBRT in patients with early stage breast cancer treated with chemotherapy. However, single-center retrospective analyses of small numbers of patients have indicated that patients who receive chemotherapy may have a higher risk for recurrence (21, 47) possibly related to the specific molecular typing of their breast cancer; therefore, the relationship between chemotherapy and IORT needs to be further investigated.

Not all patients are suitable for IORT treatment, and it is uncertain whether patients with BC with lymph node metastases will benefit from IORT. The ELIOT study excluded patients with lymph node metastases (27), and the TARGIT-A study included patients with cN1, but patients with lymph node metastases in the IORT group were supplemented with conventional EBRT for 3–6 weeks because IORT alone in this high-risk group may increase the risk of recurrence and violate ethical requirements (25). Lymph node metastasis is a high-risk factor for recurrence and predicts later BC staging, and for some studies with no restriction on the number of lymph nodes involved, N-stage was shown to be significantly associated with distant metastasis-free survival rates (48); therefore, EBRT remains the best clinical option for patients with lymph node metastasis at present. However, further studies are needed to confirm the applicability of IORT to a subgroup of people with lymph node metastases, and ethical limitations are a major impediment to studying this subgroup.

This study also has some limitations. First, it was a retrospective study with a potential for selection bias, although PSM statistical methods were utilized to reduce the bias and improve the reliability of the findings. Second, in the comparison of the two different radiotherapy modalities, although recurrence is an important factor worth analyzing, this study was unable to analyze the local recurrence rate in the IORT population and the EBRT population because recurrence information is not recorded in the SEER database. However, the long-term follow-up results of the TAGIT-A study showed that the 5-year risk of local recurrence in the IORT group was only 2.11%. In addition, unfortunately, the SEER database does not provide information on the modality of intraoperative radiotherapy, irradiation dose, and body mass index, which may be relevant to survival outcomes.

The results of this study reveal that among patients with early stage breast cancer without lymph node metastases, the survival rate of the IORT population is comparable to that of the EBRT population, and that the results of several clinical trials indicate that BCS combined with IORT for early stage breast cancer is safe and effective. The future direction of IORT research may be devoted to selecting suitable populations for developing individualized treatment strategies. In addition, more prospective clinical studies with large sample sizes in populations with low recurrence rates are needed to demonstrate the comparable efficacy of IORT vs. EBRT, especially in terms of local recurrence rates. However, the value of IORT as an alternative to EBRT for patients with early stage breast cancer without lymph node metastases is now well-established.

## Conclusion

This study showed that among patients with T1-2 early stage breast cancer without lymph node metastases, there was no significant difference in OS or BCSS rates between those who received IORT after BCS and those who received EBRT. Compared to EBRT, IORT was not associated with a worse prognosis among patients with breast cancer. Moreover, IORT probably showed a greater advantage in T2-stage tumors and in the subgroup that had received chemotherapy. Therefore, IORT may be a reasonable alternative to EBRT for patients with low-risk early stage breast cancer.

## Data availability statement

Publicly available datasets were analyzed in this study. This data can be found here: https://seer.cancer.gov/.

## Author contributions

WY, QZ, and QC: study design and idea construction. LQ, YH, JX, YD, and QZ: data collection and crosscheck. QC and QZ: statistical analysis and data visualization. QZ, LQ, YH, JX, YD, QC, and WY: manuscript drafting and revision. All authors cooperated in this study and read and approved the final version of the manuscript and contributed to the article and approved the submitted version.

## Footnotes

1
http://www.seer.cancer.gov

2
https://seer.cancer.gov/seerstat/

3
https://www.r-project.org/

## Supplementary material

Supplementary Figure 1 **(A)** Overall survival (OS) and **(B)** breast cancer-specific survival (BCSS) rate Kaplan–Meier curves of patients with breast cancer treated with IORT, EBRT, or no radiation. Log-rank tests determined the *P*-values.

Supplementary Figure 2 **(A)** Overall survival (OS) and **(B)** breast cancer-specific survival (BCSS) rate Kaplan–Meier curves for patients with breast cancer with stage T1 or T2 disease. *P*-values were determined by the log-rank test.

## Abbreviations

AJCC: American Joint Committee on Cancer
APBI: accelerated partial breast irradiation
BCS: breast-conserving surgery
BCSS: breast cancer-specific survival
BT: brachytherapy
CI: confidence intervals
EBRT: external beam radiotherapy
ELIOT: electron beam intraoperative radiotherapy
ER: estrogen receptor
HER2: human epidermal growth factor receptor 2
HR: hormone receptor
HRs: hazard ratios
IORT: intraoperative radiotherapy
IMRT: intensity-modulated radiotherapy
MpBC: metaplastic breast carcinoma
NSM: nipple-sparing mastectomy
OS: overall survival
PR: progesterone receptor
PSM: propensity score matching
SEER: surveillance, epidemiology, and end results
TM: total mastectomy
WBI: whole breast irradiation
3DCRT: 3D conformal radiotherapy.
