# Supplementary figures and images for: Prognosis comparison between intraoperative radiotherapy and whole-breast external beam radiotherapy for T1–2 stage breast cancer without lymph node metastasis treated with breast-conserving surgery: A case–control study after propensity score matching
Source: Front Med (Lausanne). 2022 Aug 3;9:919406. doi: 10.3389/fmed.2022.919406 (PMC9381880; doi:10.3389/fmed.2022.919406)

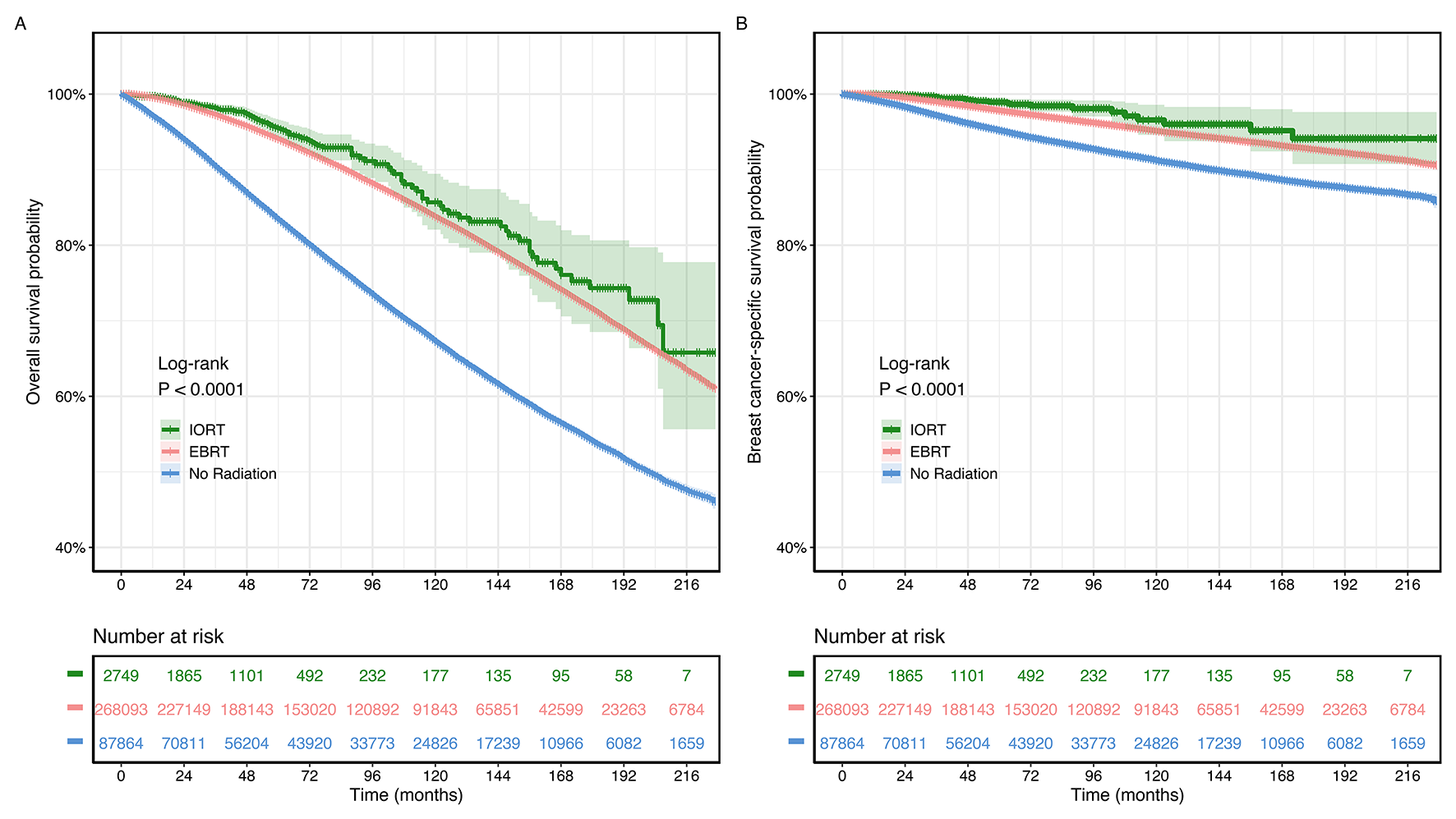

Supplement: Supplementary Figure 1 — (A) Overall survival (OS) and (B) breast cancer-specific survival (BCSS) rate Kaplan–Meier curves of patients with breast cancer treated with IORT, EBRT, or no radiation. Log-rank tests determined the P-values. [file Image_1.TIFF]

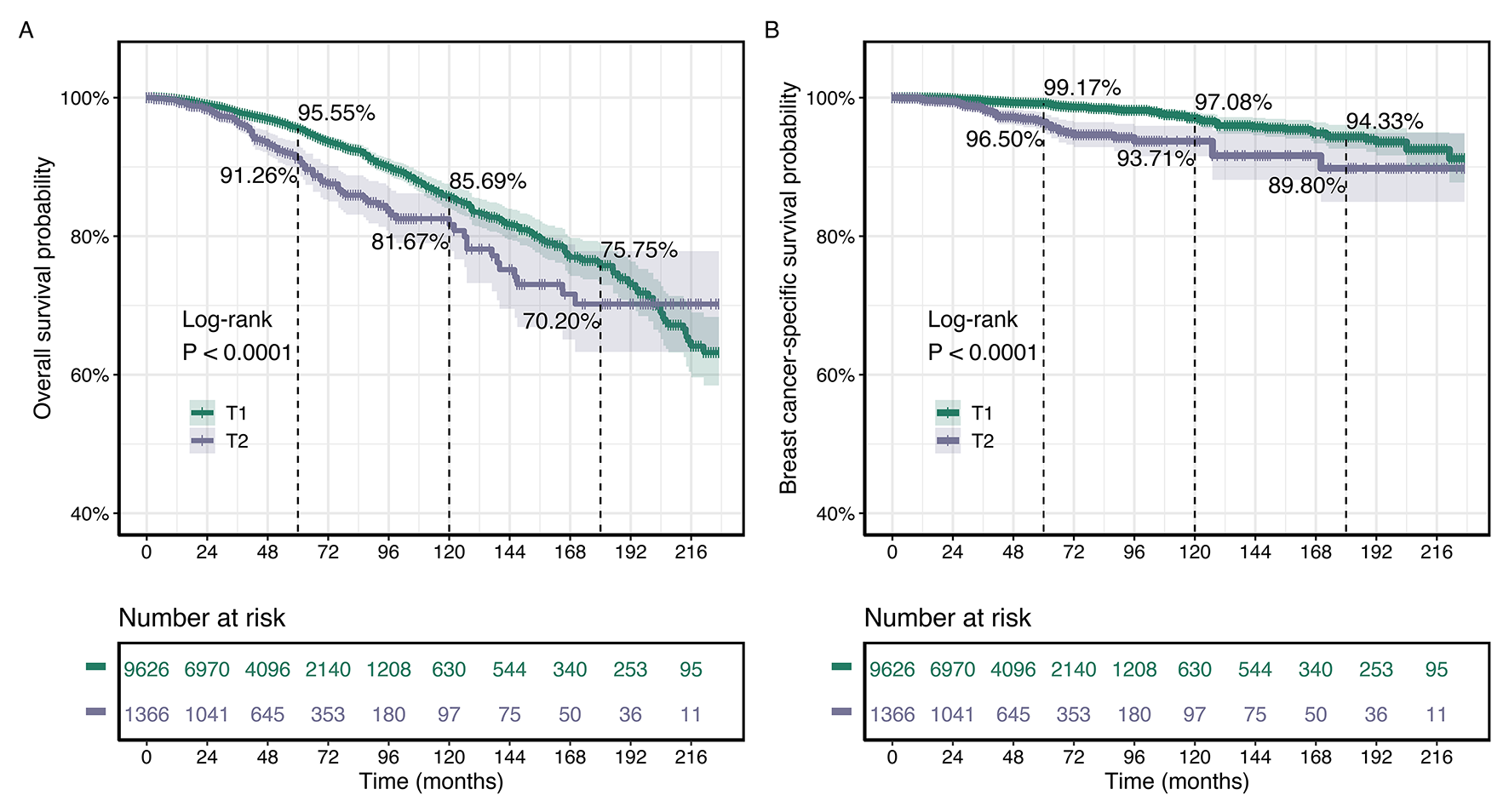

Supplement: Supplementary Figure 2 — (A) Overall survival (OS) and (B) breast cancer-specific survival (BCSS) rate Kaplan–Meier curves for patients with breast cancer with stage T1 or T2 disease. P-values were determined by the log-rank test. [file Image_2.TIFF]
