# Supplementary material for: Prognosis comparison between intraoperative radiotherapy and whole-breast external beam radiotherapy for T1–2 stage breast cancer without lymph node metastasis treated with breast-conserving surgery: A case–control study after propensity score matching
Source: Front Med (Lausanne). 2022 Aug 3;9:919406. doi: 10.3389/fmed.2022.919406 (PMC9381880; doi:10.3389/fmed.2022.919406)
Supplement: Supplementary file 3 [file Data_Sheet_1.PDF]

Supplementary Table 1. Characteristics of breast cancer patients without lymph node metastasis at stage T1 and T2 in the SEER database

Category	Overall<br>10992	T1<br>9626	T2<br>1366	*P* - value
Age				<0.001
≤45	288 (2.6)	228 (2.4)	60 (4.4)	
46-65	5407 (49.2)	4740 (49.2)	667 (48.8)	
>65	5297 (48.2)	4658 (48.4)	639 (46.8)	
Year				<0.001
2000–2004	523 (4.8)	447 (4.6)	76 (5.6)	
2005–2009	383 (3.5)	330 (3.4)	53 (3.9)	
2010–2013	2479 (22.6)	2091 (21.7)	388 (28.4)	
2014–2018	7607 (69.2)	6758 (70.2)	849 (62.2)	
Marital status				0.030
Married	6458 (58.8)	5689 (59.1)	769 (56.3)	
Single	1752 (15.9)	1497 (15.6)	255 (18.7)	
DSW	2443 (22.2)	2143 (22.3)	300 (22.0)	
Unknown	339 (3.1)	297 (3.1)	42 (3.1)	
Race				<0.001
White	9030 (82.2)	7921 (82.3)	1109 (81.2)	
African American	789 (7.2)	653 (6.8)	136 (10.0)	
Other	1091 (9.9)	972 (10.1)	119 (8.7)	
Unknown	82 (0.7)	80 (0.8)	2 (0.1)	
Histology				<0.001
Ductal carcinoma	8734 (79.5)	7743 (80.4)	991 (72.5)	
Lobular carcinoma	1145 (10.4)	929 (9.7)	216 (15.8)	
Other	1113 (10.1)	954 (9.9)	159 (11.6)	
Grade				<0.001
I	4453 (40.5)	4136 (43.0)	317 (23.2)	
II	5032 (45.8)	4293 (44.6)	739 (54.1)	
III	1356 (12.3)	1049 (10.9)	307 (22.5)	
IV	18 (0.2)	18 (0.2)	0 (0.0)	
Unknown	133 (1.2)	130 (1.4)	3 (0.2)	
ER				<0.001
Positive	10350 (94.2)	9112 (94.7)	1238 (90.6)	
Negative	545 (5.0)	428 (4.4)	117 (8.6)	
Unknown	97 (0.9)	86 (0.9)	11 (0.8)	
PR				0.001
Positive	9497 (86.4)	8361 (86.9)	1136 (83.2)	
Negative	1397 (12.7)	1181 (12.3)	216 (15.8)	
Unknown	98 (0.9)	84 (0.9)	14 (1.0)	
HER2				0.265
Positive	416 (3.8)	362 (3.8)	54 (4.0)	
Negative	9420 (85.7)	8271 (85.9)	1149 (84.1)	
Unknown	248 (2.3)	216 (2.2)	32 (2.3)	
Unavailable	908 (8.3)	777 (8.1)	131 (9.6)	
Molecular Subtype				<0.001
HR+/HER2-	9138 (83.1)	8049 (83.6)	1089 (79.7)	
HR+/HER2+	358 (3.3)	314 (3.3)	44 (3.2)	
HER2 enriched	58 (0.5)	48 (0.5)	10 (0.7)	
TNBC	280 (2.5)	220 (2.3)	60 (4.4)	
Unknown	1158 (10.5)	995 (10.3)	163 (11.9)	
Chemotherapy				<0.001
Chemotherapy	1136 (10.3)	854 (8.9)	282 (20.6)	
Chemotherapy-naïve/Unknown	9856 (89.7)	8772 (91.1)	1084 (79.4)	
Radiation Sequence				1.000
IORT	2749 (25.0)	2407 (25.0)	342 (25.0)	
EBRT	8243 (75.0)	7219 (75.0)	1024 (75.0)	

Abbreviations: DSW, divorced/separated/widowed; EBRT, external beam radiotherapy; ER, estrogen receptor; HER2, human epidermal growth receptor 2; HR, hormone receptor; IORT, intraoperative radiotherapy; PR, progesterone receptor; TNBC, triple-negative breast cancer.
