# Supplementary material for: Prognosis comparison between intraoperative radiotherapy and whole-breast external beam radiotherapy for T1–2 stage breast cancer without lymph node metastasis treated with breast-conserving surgery: A case–control study after propensity score matching
Source: Front Med (Lausanne). 2022 Aug 3;9:919406. doi: 10.3389/fmed.2022.919406 (PMC9381880; doi:10.3389/fmed.2022.919406)
Supplement: Supplementary file 4 [file Data_Sheet_2.PDF]

Supplementary Table 2. Characteristics of female patients diagnosed with T2 stage

Variables	After propensity score matching, n (%)			
	Overall	IORT	EBRT	*P*-value
Age				0.801
≤45	60 (4.4)	13 (3.8)	47 (4.6)	
46-65	667 (48.8)	170 (49.7)	497 (48.5)	
>65	639 (46.8)	159 (46.5)	480 (46.9)	
Year				0.986
2000–2004	76 (5.6)	19 (5.6)	57 (5.6)	
2005–2009	53 (3.9)	14 (4.1)	39 (3.8)	
2010–2013	388 (28.4)	99 (28.9)	289 (28.2)	
2014–2018	849 (62.2)	210 (61.4)	639 (62.4)	
Marital status				0.954
Married	769 (56.3)	188 (55.0)	581 (56.7)	
Single	255 (18.7)	66 (19.3)	189 (18.5)	
DSW	300 (22.0)	77 (22.5)	223 (21.8)	
Unknown	42 (3.1)	11 (3.2)	31 (3.0)	
Race				0.806
White	1109 (81.2)	274 (80.1)	835 (81.5)	
African American	136 (10.0)	35 (10.2)	101 (9.9)	
Other	119 (8.7)	32 (9.4)	87 (8.5)	
Unknown	2 (0.1)	1 (0.3)	1 (0.1)	
Histology				0.935
Ductal carcinoma	991 (72.5)	249 (72.8)	742 (72.5)	
Lobular carcinoma	216 (15.8)	55 (16.1)	161 (15.7)	
Other	159 (11.6)	38 (11.1)	121 (11.8)	
Grade				0.353
I	317 (23.2)	75 (21.9)	242 (23.6)	
II	739 (54.1)	185 (54.1)	554 (54.1)	
III	307 (22.5)	80 (23.4)	227 (22.2)	
Unknown	3 (0.2)	2 (0.6)	1 (0.1)	
ER				0.868
Positive	1238 (90.6)	311 (90.9)	927 (90.5)	
Negative	117 (8.6)	29 (8.5)	88 (8.6)	
Unknown	11 (0.8)	2 (0.6)	9 (0.9)	
PR				0.954
Positive	1136 (83.2)	284 (83.0)	852 (83.2)	
Negative	216 (15.8)	54 (15.8)	162 (15.8)	
Unknown	14 (1.0)	4 (1.2)	10 (1.0)	
HER2				0.514
Positive	54 (4.0)	18 (5.3)	36 (3.5)	
Negative				
Unknown	1149 (84.1)	282 (82.5)	867 (84.7)	
Unavailable	32 (2.3)	9 (2.6)	23 (2.2)	
Molecular Subtype	131 (9.6)	33 (9.6)	98 (9.6)	
HR+/HER2-				0.689
HR+/HER2+	1089 (79.7)	267 (78.1)	822 (80.3)	
HER2 enriched	44 (3.2)	15 (4.4)	29 (2.8)	
TNBC	10 (0.7)	3 (0.9)	7 (0.7)	
Unknown	60 (4.4)	15 (4.4)	45 (4.4)	
Chemotherapy				0.987
Chemotherapy	163 (11.9)	42 (12.3)	121 (11.8)	
Chemotherapy-naïve	282 (20.6)	70 (20.5)	212 (20.7)	
**Total**	1084 (79.4)	272 (79.5)	812 (79.3)	

Abbreviations: DSW, divorced/separated/widowed; EBRT, external beam radiotherapy; ER, estrogen receptor; HER2, human epidermal growth receptor 2; HR, hormone receptor; IORT, intraoperative radiotherapy; PR, progesterone receptor; TNBC, triple-negative breast cancer.
